# The Extremes of Constipation: A Case of Stercoral Perforation From Fecal Impaction in a Teenager

**DOI:** 10.7759/cureus.43554

**Published:** 2023-08-16

**Authors:** Felicia Lee, Jasmin Cao, Evan Lin, Maho Kurashima, Raymond I Okeke, Christian Saliba, Shin Miyata

**Affiliations:** 1 Medical School, Saint Louis University School of Medicine, Saint Louis, USA; 2 Pediatric Surgery, SSM Health Cardinal Glennon Children’s Hospital, Saint Louis, USA; 3 General Surgery, SSM Health Saint Louis University Hospital, Saint Louis, USA

**Keywords:** perforation, colostomy, constipation, colitis, stercoral colitis

## Abstract

Stercoral perforation is a rare sequela of poorly controlled constipation that is more commonly seen in older, bedridden patients than in pediatric patients. We present the case of a 13-year-old patient requiring a divided sigmoid colostomy following rectal perforation, one of the few examples in the pediatric literature of stercoral perforation from chronic constipation. The current report highlights the importance of appropriate treatment of functional constipation at onset and the life-threatening complications that can occur without appropriate follow-up.

## Introduction

Chronic idiopathic constipation affects up to 30% of the pediatric population [1-3]. Also known as functional constipation, it is defined as the accumulation of large, hard stool from infrequent bowel movements that can result in painful defecation and fecal incontinence [4]. It should be differentiated from pathological causes of constipation, including Hirschsprung’s disease, which typically occurs in the first few weeks of life and is diagnosed by the absence of ganglion cells in a rectal biopsy; however, it can be missed during the neonatal period and present as severe constipation later on. The management of functional constipation in the pediatric population includes the use of nonpharmacologic measures such as sorbitol-containing juices and medications including lactulose, polyethylene glycol, and suppositories. Stercoral perforation is a feared and extremely rare complication of chronic constipation more commonly seen in older, bedridden patients. There are very few case studies reporting stercoral perforation from chronic constipation in younger patients [5]. Here, we present the case of a 13-year-old female with a history of chronic constipation that was complicated by rectal perforation and treated by surgical intervention with the creation of a diverting sigmoid colostomy and mucous fistula.

## Case presentation

**Figure 1:**
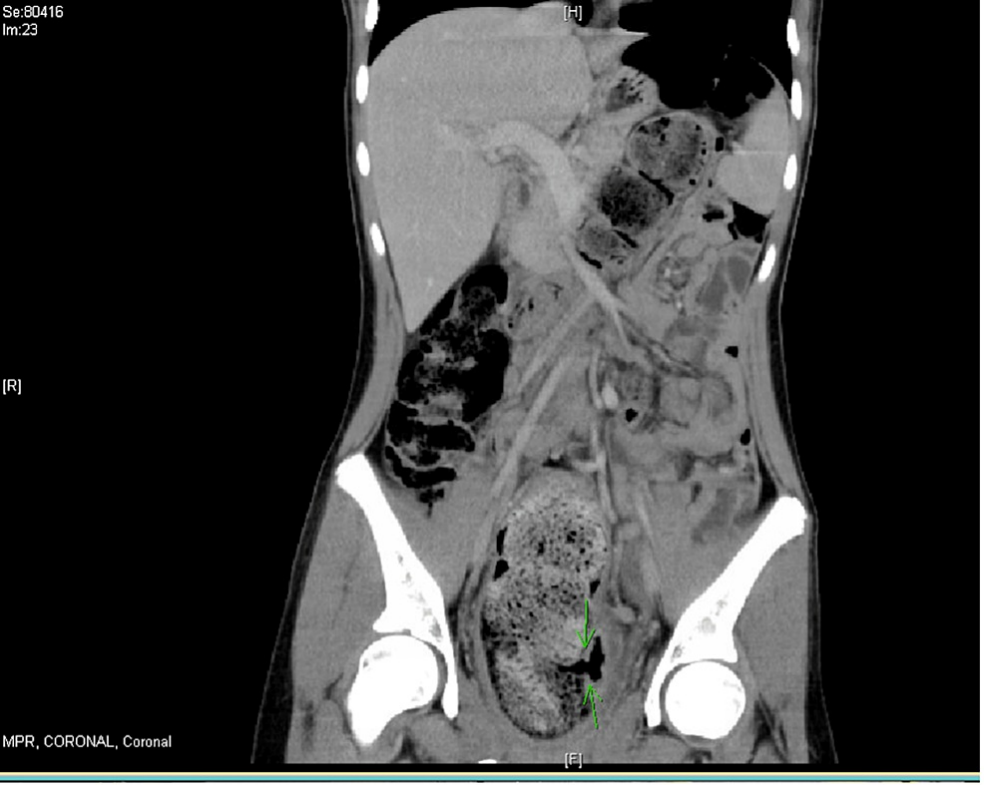
Coronal computed tomography imaging showing the site of rectal perforation (green arrows).

**Figure 2:**
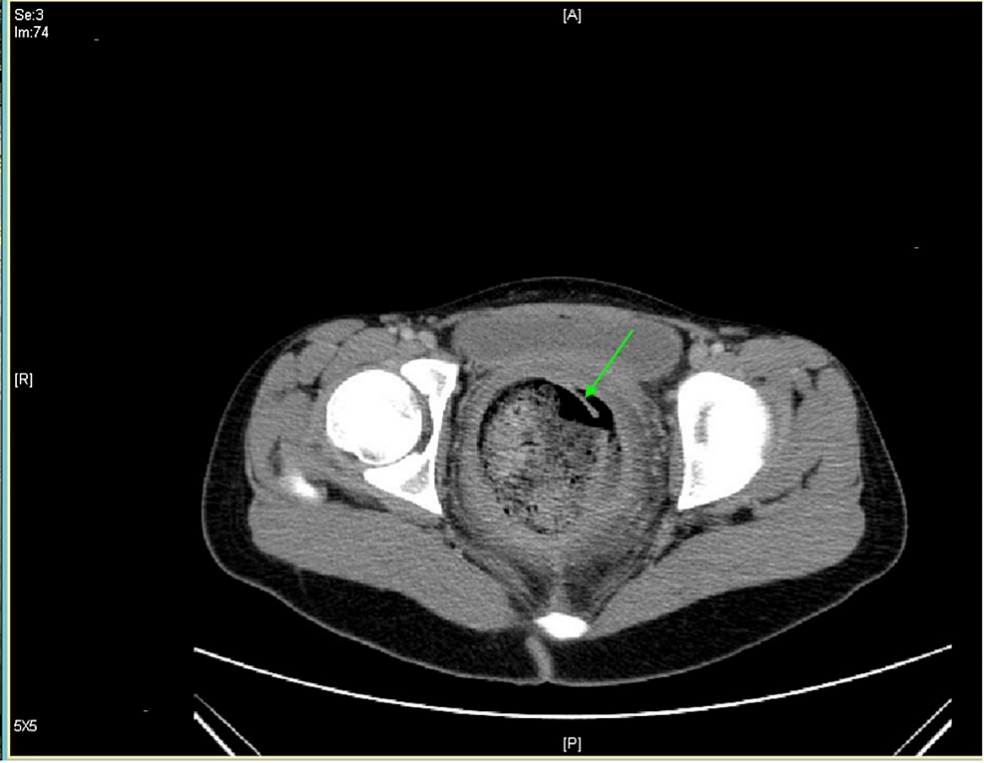
Sagittal computed tomography imaging showing rectal perforation (green arrow).

A 13-year-old female with no significant past medical history except for chronic constipation presented to the emergency room with severe right lower quadrant (RLQ) pain and obstipation. On arrival, she was hypotensive at 70/30 mmHg and was tachycardic at 110 beats per minute. She had a normal respiratory rate and saturation on room air. The patient was alert but appeared paler than typical according to her mother. The abdominal examination was notable for firmness and tenderness to palpation at the RLQ area. There was no tenderness to palpation in other areas of her abdomen but stool burden was palpable. Bowel sounds were also present throughout. Cardiac, lung, and skin examinations were unremarkable. Complete blood count and metabolic panels were unremarkable with a white blood cell count (WBC) of 10,300 WBCs per microliter. She was administered 3 L of normal saline boluses and started on a norepinephrine drip and a piperacillin and tazobactam infusion. Computed tomography (CT) imaging from the referring institution demonstrated rectal wall discontinuity at the 2 o’clock position with stool leakage and a large stool burden in the descending colon, sigmoid colon, and rectum (Figures 1, 2). There was no evidence of free air. A normal appendix was visualized. The findings concerned for contained perforation of the lateral rectal wall.

The patient was urgently taken to the operating room for rectal examination under anesthesia, diagnostic laparoscopy, rectal biopsy, repair of rectal perforation, and creation of a sigmoid ostomy. The rectum was initially examined with a digital rectal examination and anoscopy which demonstrated a 5 mm × 3 cm perforation at the 2 o’clock position on the rectal wall and a 1 cm × 0.5 cm defect of the mucosa at the 6 o’clock position. Given an enlarged abscess cavity seen outside of the 2 o’clock perforation, a red rubber catheter was placed at the site for drainage and secured to the rectal wall with an absorbable suture. On diagnostic laparoscopy, the catheter was not visible inside the peritoneum, confirming that this site was an extraperitoneal perforation. In addition, no intraperitoneal stool contamination was seen. A biopsy of the rectum was then obtained 2 cm proximal to the dentate line, adjacent to the 6 o’clock position. A laparoscopic-assisted diverting sigmoid colostomy was created at the descending sigmoid junction in the standard fashion. There were no complications during the procedure. Biopsy results showed necrotic colorectal mucosa likely from pressure and the presence of normal ganglion cells with no other pertinent findings.

The patient did well post-surgery and her bowel function normalized by postoperative day three. She was discharged on postoperative day four on a bowel regimen. The drain fell out on its own three weeks after discharge. A follow-up examination under anesthesia and contrast enema two months after the index procedure demonstrated a well-healed rectal wall without stricture. A colostomy takedown was planned after an interval of nutritional optimization.

Six months after examination under anesthesia, she underwent a scheduled colonoscopy and motility evaluation in preparation for an ostomy takedown that demonstrated colitis in the rectosigmoid region. However, the workup for inflammatory bowel disease, including biopsy, was unremarkable and the inflammation was thought to be due to diversion colitis. A laparoscopic sigmoidectomy during colostomy takedown was performed to prevent recurrence by resecting the redundant colon. She recovered well postoperatively and was discharged home.

## Discussion

According to the American College of Gastroenterology, constipation accounts for at least 2.5 million doctor visits per year [6]. Chronic constipation may lead to severe fecal impaction which increases colonic wall pressure and distention and can cause serious consequences such as bowel ischemia and perforation [6,7]. Most patients with stercoral colitis present with abdominal pain, cramps, and fever, with a history of poorly managed chronic constipation [1,7]. The most important differential diagnosis to be ruled out for severe constipation is Hirschsprung’s disease, especially in the pediatric population. Physical examination may reveal abdominal tenderness and distension, as well as the presence of stool impaction in the rectum. Stercoral colitis can lead to complications such as perforations and bowel ischemia, in which patients may present with signs of peritonitis, sepsis, or shock [8]. Perforation is an uncommon but fatal complication of stercoral colitis with a high mortality rate of 30-40% [6]. It is particularly rare in children due to the increased flexibility of the colonic wall in children compared to adults. Interestingly, pediatric patients can often present with nonspecific findings or present similarly to appendicitis, such as in our patient with RLQ pain, masking the diagnosis of stercoral colitis and perforation [5]. Thus, early imaging is necessary for prompt detection, accurate diagnosis, and a favorable prognosis in these cases. CT imaging of the abdomen and pelvis is the gold standard for diagnosing stercoral perforation, with extraluminal gas bubbles and abscess formation typically indicating the progression of stercoral colitis to perforation [8].

Management of patients with uncomplicated bowel obstruction and absent signs of peritonitis is often nonoperative, including manual or endoscopic removal of fecal impaction, aggressive bowel regimen, and observation [9]. In cases of sepsis or peritonitis, intravenous antibiotics and fluids are administered [8,9]. For patients who have failed conservative therapy or those with complications such as stercoral perforation and bowel ischemia, surgical intervention is the only effective treatment modality [6]. The affected bowel segments may be resected and anastomosed with possible ostomy creation, such as in the case of our patient. Patients are also started on broad-spectrum antibiotics and closely monitored [1,8]. All patients with constipation should be educated on the importance of lifestyle modifications such as increasing fiber and fluid consumption, the addition of sorbitol-containing juices, as well as the management of their condition through medications such as laxatives and suppositories to prevent bowel obstruction and its potentially life-threatening complications [1,10].

## Conclusions

Stercoral perforation is a rare but serious complication of poorly controlled constipation that can result in sepsis and potentially lead to a lethal outcome. Our presented case involves a stercoral rectal perforation in a pediatric patient due to chronic constipation. This case emphasizes the significance of implementing appropriate treatment for functional constipation, performing a full workup to rule out pathological etiologies of constipation such as Hirschsprung’s disease, and conducting an early evaluation of possible perforation.
